# Eastern asian expert panel opinion: designing clinical trials of molecular targeted therapy for hepatocellular carcinoma

**DOI:** 10.1186/1471-2407-10-620

**Published:** 2010-11-10

**Authors:** Winnie Yeo, Pei-Jer Chen, Junji Furuse, Kwang-Hyub Han, Chiun Hsu, Ho-Yeong Lim, Hanlim Moon, Shukui Qin, Ee-Min Yeoh, Sheng-Long Ye

**Affiliations:** 1 Prince of Wales Hospital, Shatin, Hong Kong; 2 National Taiwan University Hospital, Taipei, Taiwan; 3 Kyorin University Hospital, Tokyo, Japan; 4 Yonsei University, College of Medicine, Seoul, South Korea; 5 Samsung Medical Centre, Seoul, South Korea; 6 GlaxoSmithKline, Singapore; 7 No. 81 Hospital of PLA, Nanjing, China; 8 Zhongshan Hospital, Shanghai, China

## Abstract

The largest burden of hepatocellular carcinoma (HCC) lies in Asia, secondary to hepatitis B virus (HBV) infection. Improved survival with sorafenib has fostered new research but many challenges remain in designing clinical trials. The disease, its management, and populations affected by it are heterogeneous worldwide and within Asia. An expert conference of Eastern Asian oncologists and hepatologists was convened to foster consensus in clinical trial design. The panel identified key areas that need to be addressed to facilitate clinical trials in Asia. Stratification by viral etiology is desirable within Asia and by region in global trials. Antiviral therapy should also be considered as a stratification factor and incorporated into HCC management in trials. The panel agreed that histological diagnosis is not required for trial entry and that Barcelona-Clinic Liver Cancer (BCLC) staging is acceptable for trials as long as portal hypertension can be better defined with standardized methodology. Consensus in treatment must be sought to allow multi-national trials and it must be recognized that first-line sorafenib is not largely feasible in Asia. Finally, Asian nations must be urged to participate in clinical trials, many of which are ongoing, to advance new treatment options in this challenging disease.

## Background

Over 600,000 cases of hepatocellular carcinoma (HCC) are diagnosed annually worldwide and the mortality-to-incidence rate ratio is second only to pancreatic cancer [1,2]. The incidence of HCC varies widely by geographical region. Asia carries the largest burden with 55% of all cases occurring in China [1]. Age-standardized incidence rates per 100,000 persons for men are 45.0 in Korea (1999-2001) [3], 37.9 in China (2002) [1], and 23.1 in Japan (2002) [1]. Corresponding rates for women are 12.0, 14.2, and 7.6. Globally, the predominant cause of HCC is viral infection with hepatitis B virus (HBV) or hepatitis C virus (HCV) [4].

Hepatocellular carcinoma is refractory to cytotoxic chemotherapy [5] and the failure of cytotoxic regimens has led to a bleak outlook. However, the recent development of molecular targeted therapies is changing the landscape and offering hope. Researchers have found new optimism for initiating clinical trials after sorafenib showed efficacy in advanced disease [6]. Currently, trials are planned or ongoing in all stages of HCC; however, many issues remain [7]. Most salient is the variability in management practices both between Asia and the West and within Asia. Key differences are apparent in the etiology, diagnosis, staging, and treatment of HCC among countries. These differences complicate the conduct of international clinical trials that will foster approval and availability of new therapeutic entities.

In order to forge a better understanding of how HCC clinical practices in the Eastern Asian region compare to current global clinical trial requirements, an expert conference was held. Participants of the panel (the authors) are oncologists and hepatologists representing China, Hong Kong, Japan, Korea, and Taiwan who have an expertise in treating HCC. Each panelist offered insight, reviewed herein, about how HCC is managed across Eastern Asia and how management practices and clinical trial requirements can be unified to advance new treatments, particularly targeted agents, for HCC.

## Etiology

Viral etiology varies by region with HBV predominating in non-Japanese Asians and accounting for approximately 70-80% of cases. In Japan, most of Europe, and in the United States, HCV is more common than HBV among viral etiologies [3,8-11]. However, in the United States, 67% of HCC cases are seronegative for both viruses [10].

The increased incidence of HBV-HCC in Eastern Asia compared to Japan and Western nations leads to different management issues and prognosis that affect clinical trial design. Hepatitis C virus-HCC is more likely to develop in the background of cirrhosis than HBV-HCC [12]. Therefore, the underlying liver disease may differ in HCC patients by region, a factor that weighs heavily in treatment decisions.

Survival differences have been observed according to geographic region and viral etiology, though the reasons for these observations remain unclear. In clinical trials of systemic therapy for advanced HCC, trials done in Asian countries reported inferior survival compared with trials done in non-Asian countries [13]. Possible reasons include variation in genetic and/or epigenetic aberrations between different viral etiologies and the propensity for Asian physicians to use local therapy more aggressively and in later stages, resulting in enrollment of a more advanced patient population to trials of systemic therapy. Survival between HBV-HCC and HCV-HCC appears similar in early-stage, resectable HCC, if staging and other clinical parameters are considered [14]. However, two retrospective studies have found poorer survival in HBV-HCC among patients with unresectable, advanced disease [15,16]. Attributing the survival difference to viral etiology alone is difficult but demonstrates the need for considering the potential differences in clinical trials.

Additionally, in contrast to HCV, HBV reactivates with immune suppression, complicating treatment with immunosuppressive regimens [17,18]. The predominance of HBV-HCC in Asia is associated with increased use of antiviral agents to prevent viral reactivation during HCC treatment. Antiviral therapy with lamivudine has reduced the incidences of HBV reactivation and hepatitis, reduced the severity of hepatitis episodes, led to fewer disruptions in chemotherapy, and reduced mortality related to HBV reactivation in clinical trials of patients with HCC or other cancers who are receiving chemotherapy [19-22]. Anti-viral therapy following curative resection, radiofrequency ablation, or other local, non-chemotherapeutic treatments for HBV-HCC, has been shown to increase residual liver volume and/or function and may prolong survival [23-25]. Furthermore, interferon, given after curative therapy, may increase recurrence-free survival rates [26,27]. These benefits indicate that use of antiviral therapy is an important confounding factor in HCC clinical trials.

A separate international expert panel has recommended stratification according to region for global trials but discouraged further stratification according to etiology [7]. However, in light of the confounding factors described herein, the current panel agreed that trials within Eastern Asia should include stratification by HBV or HCV etiology. Further, antiviral therapy should be both considered as a stratification factor and incorporated into the overall management of patients in international HCC clinical trials.

## Screening

Stage at diagnosis differs both within Eastern Asia and between Eastern Asia and Western nations. Using TNM-based staging systems, China and Japan have relatively high proportion of patients diagnosed at Stage I or II compared to Hong Kong and Korea. In the United States, a higher percentage of patients are diagnosed with distant metastasis compared to Asian countries [28,29].

The differences may reflect variable screening practices. The proportion of patients who receive screening in the United States appears to vary according to the individual's healthcare. Only 25% of family practice physicians report routinely screening appropriate patients for HCC compared to 84% of physicians who are members of the Association for the Study of Liver Diseases (AASLD) [30,31]. In a study of 157 patients diagnosed with HCC at three US Veteran Affairs (VA) medical centers, 39% of patients with a known risk factor for HCC received screening [32]. With the exception of Hong Kong, where screening has been conducted in the context of study, screening high-risk populations is the standard of care in Asia. With diagnosis occurring at earlier stages, Eastern Asian countries are better able to utilize curative therapies, significantly affecting treatment paradigms and clinical trial populations.

## Diagnosis

Both pathological and clinical diagnostic procedures vary according to country. The majority of pathological diagnoses are made by core biopsy in Korea, China, and Hong Kong, with fine needle aspiration (FNA) used infrequently. In contrast, 30% or fewer of pathological diagnoses are made by core biopsy in Japan, and Taiwan. Taiwan employs FNA in approximately 10% of cases but utilizes surgery for pathologic diagnosis in approximately 38% of cases. Protocols designating biopsy-proven HCC as an enrollment requirement would conflict with current practices in Japan and Taiwan. The panel agreed that for trials conducted in the advanced/metastatic setting, histological confirmation of HCC is not necessary. Further, pre-treatment biopsy may result in tumor seeding which would complicate neoadjuvant trials.

## Staging

A variety of staging systems are employed worldwide [33-36]. Several of these systems are based on the tumor-node-metastasis (TNM) paradigm or incorporate TNM groupings as a variable [33-35,37]. Other systems, such as the Barcelona-Clinic Liver Cancer (BCLC) staging system, incorporate measures of liver function and underlying disease. Complicating international clinical trial design is the variable use of these systems both within Asia and globally. Each region of Asia represented by the panel currently utilizes a different system. In China, the revised Staging Criteria of Primary Liver Cancer is used. This system was developed by the Chinese Society of Liver Cancer. The system uses criteria based on size, number and location of tumors, lymph node spread, extrahepatic metastasis, portal vein thrombosis, and liver function (Child-Pugh scores) [38]. In Japan, both the staging system and treatment algorithm apply liver function as the first category of evaluation rather than tumor size. Hong Kong does not have a unified staging system. Although BCLC is considered a valuable tool for a treatment algorithm in Hong Kong, the system is considered less useful for prognostication. The Chinese University Prognostic Index (CUPI) [37] has been found useful for prognostication at one center due to the more advanced population [39]. Korea employs a modified International Union Against Cancer (UICC) system and Taiwan uses BCLC.

The TNM-based staging systems have an important drawback: these systems do not account for underlying liver disease [40]. In HCC, the presence of liver disease is a common and important prognostic factor that is integral in determining treatment [40,41]. For these reasons, TNM-based systems have limited value in the comprehensive management of HCC. The Child-Pugh (CP) score is a widely-accepted system to evaluate liver function. Despite empirical selection of variables, this tool represents a simple, bedside tool that predicts mortality in cirrhotic patients with a degree of accuracy not substantially less than the more statistically sound model for end-stage liver disease (MELD) [42]. The BCLC staging system incorporates measures of liver function (portal hypertension, bilirubin, and CP scores at higher stages) and has emerged as the standard for clinical trial design [6,43]. However, this system is not generally used in Eastern Asia with the exception of Taiwan. China, specifically, has failed to adopt this system due to the omission of portal vein thrombosis as a factor, which has been shown to independently predict mortality [41]. Additionally, BCLC includes portal venous hypertension which requires an invasive procedure to measure that is not standard practice in Asia. However, the panel indicated that, if required for clinical trials seeking United States Food and Drug Administration approval, BCLC would be acceptable if the protocols also incorporated portal vein hypertension - measured and defined with non-invasive standardized methodology - and further evaluation of liver function.

## Treatment Practices

Treatment practices vary somewhat throughout Eastern Asia and no unified treatment algorithm exists. Japan, China, Hong Kong, Korea, and Taiwan each use separate treatment algorithms, all of which differ from the BCLC treatment algorithm [7,44,45]. Such variations in treatment practices cause challenges in defining treatment protocols for international clinical trials.

### Potentially Curative Treatment Options

Resection is utilized more often in Eastern Asia versus Western nations, which may reflect diagnosis at earlier stages and less cirrhosis in Asia [46]. In some centres in China, Taiwan, and Japan, between 34-40% of patients undergo resection, while the proportion is approximately 10-20% in others. In parts of East Asia [47,48], patients with recurrence undergo re-resection. Local ablation is performed in approximately 15% of patients in China, Hong Kong, and Taiwan and approximately 30% of patients in Japan. Liver transplant is the only treatment modality that offers a cure both for HCC and the underlying liver disease, but its application is limited both in Eastern Asia and the West.

### Nonsurgical Local Treatments

Although TACE and transarterial embolization (TAE) are standards of care, significant heterogeneity exists among countries and institutions with respect to the types of embolizing materials and techniques utilized. Embolizing materials used typically include a mixture of iodized oil (lipiodol) and an anthracycline (epirubicin or doxorubicin) or cisplatin followed by gelatin sponge particles (Japan, Taiwan, Hong Kong). Nonetheless, other agents are used, particularly in China where 5-fluorouracil (5-FU) and mitomycin-C may be employed. Japan uses HAI with cisplatin alone, 5-FU and cisplatin (FP), or 5-FU and interferon. Currently, no consensus has been reached regarding the interval between procedures or endpoints. Other local therapies are variably utilized and include intratumoral injection, laser therapy, cryotherapy, microwave coagulation therapy, hepatic arterial infusion (HAI), intraarterial radiotherapy with yttrium-90 and conformal external radiotherapy.

### Systemic Therapy With Sorafenib

Targeted therapy has been employed only for advanced disease [7,44,45]. A multitude of targeted therapies have been investigated for use in HCC; however, only sorafenib is approved for use in Asian and Western countries. These approvals were based on improved survival in the SHARP trial and the parallel Asian phase III trial [6,49]. Although sorafenib has been approved in Asia, the agent is not widely used largely due to cost [50]. Cost-sharing programs have been started in some countries to manage this issue. Such programs have been successful in that they expand usage; however, lack of long-term coverage renders the practice unsustainable.

In addition to cost, emerging evidence suggests that sorafenib may be less well tolerated by Asian patients compared to Western patients. Hand-foot skin reaction (HFSR) appears to be more frequent in Asians, particularly lower-grade reactions. Hand-foot skin reaction (all grades) occurred in 21% of patients in the US SHARP study; the rate was 45% in the Asian phase III sorafenib trial [6,46]. Grade 3 event rates were 8% in SHARP compared with 11% in the Asian trial. Korean and Japanese studies have reported rates of 56%-57% (all grades) [51,52]. In the Korean population, HFSR was the most common reason for treatment interruption. Indeed, dose reductions for HFSR were more frequent in the Asian phase III trial (11%) than in SHARP (5%) [6,46] The panelists noted that in practice, dose reduction or use of a reduced starting dose of sorafenib is common in Asia. Lower dosing is being investigated in small Asian trials. In a Japanese phase I study, sorafenib 200 mg twice daily led to a 38% incidence of HFSR [52].

Though HFSR is most common, some differences between Westerners and Asians may be present with respect to the drug's effect on the liver. The Korean population experienced a 4% rate of grade 3 or 4 hyperbilirubinemia associated with marked ALT elevations [51]. Individual differences in drug metabolism may be present. Increased bilirubin was reported separately in a patient with UGT1A1 polymorphism; the authors proposed that sorafenib inhibition of UGT1A1 in this patient may have contributed to the hyperbilirubinemia [53].

### Other Systemic Therapies

Systemic cytotoxic chemotherapy has failed to prolong survival in advanced HCC [5]. Small studies of cytotoxic chemotherapy plus biochemical modulation may achieve tumor control in patients with good performance status and liver function reserves and no hypersplenism [54-56]. In Korea, chemotherapy is used as part of concurrent chemoradiotherapy protocols at some centers. In Hong Kong, systemic cytotoxic chemotherapy is considered when a patient fails or is ineligible for anti-VEGF therapy. Chemotherapy was not recommended in Japanese treatment guidelines.

In China, use of traditional Chinese medicine (TCM) is common and unique compared to Western nations. These medicines can be categorized according to two main purposes: 1) promoting liver health and delaying cirrhosis and 2) countering the side effects of chemotherapy. Panelists indicated that the first type of TCM must be allowed in clinical trials; excluding these treatments would severely restrict enrollment. However, the second type of TCM could potentially be excluded if required.

### Investigational Targeted Therapy

Targeted agents are at the forefront of HCC clinical research. Promoting clinical trial participation in Asia is important to foster development of new drugs appropriate for this population. Recently completed phase II trials of new treatments are described below and ongoing phase II and III trials of targeted therapies in HCC are reviewed in Table 1.

**Table 1:** Ongoing Phase II/III Trials in Advanced HCC

Study Name Clinicaltrials.gov Identifier	Phase	Intervention	Setting	Location
**Advanced Disease**				

*Targeted Agents With Cytotoxic Therapy*				

NCT00832637	II	Erlotinib + gemcitabine + oxaliplatin	Prior systemic therapy allowed	US

HOG GI06-101 NCT00532441	II	Erlotinib + docetaxel	Third-line or less	US

NCT00384800	II	Thalidomide + tegafur/uracil	No prior chemotherapy	Taiwan

NCT00519688	II	Thalidomide + tegafur/uracil	No prior chemotherapy	Taiwan

NCT00862082	I/II	Sorafenib + PR104 Sorafenib	First-line	US, Asia

*Anti-VEGF Agents as Monotherapy*				

BRISK NCT00858871	III	Brivanib + placebo Sorafenib + placebo	First-line	International

NCT00825955	III	Brivanib + placebo BSC + placebo	Sorafenib failure	International

NCT00699374	III	Sunitinib Sorafenib	First-line	International

NCT00247676	II	Sunitinib	First-line	France, Korea, Taiwan

*Other Targeted Agents as Monotherapy*				

NCT00225290	III	Thalidomide Placebo	Any line Poor liver reserve	Taiwan

NCT00033462	II	Erlotinib	First- or second-line	US

NCT00077441	II	Bortezomib	First-line	US, Australia, Korea, HK

NCT00390195	I/II	Everolimus (weekly or daily)	Any line	Taiwan

NCT00920192	I/II	Foretinib	Any line	Taiwan, HK

*Combination Targeted Therapy*				

SEARCH NCT00901901	III	Sorafenib + erlotinib Sorafenib	First-line	International

NCT00881751	II	Erlotinib + bevacizumab Sorafenib	First-line	US

NCT00365391	II	Erlotinib + bevacizumab	First- or second-line	US

TCOGP-1209 NCT00971126	I/II	Thalidomide + sorafenib	First-line	Taiwan

NCT00828594	I/II	Everolimus + sorafenib Placebo + sorafenib	First-line	International

NCT00791544	I/II	AVE1642* +/- sorafenib or erlotinib	Any line	France

**Earlier-stage Disease**				

STORM NCT00692770	III	Sorafenib Placebo	Adjuvant (post-resection or -local ablation)	International
BRISK-TA NCT00908752	III	Brivanib + TACE Placebo + TACE	BCLC B	International

NCT00921531	III	Thalidomide + TACE TACE	BCLC A-B	China

NCT00728078	II/III	Thalidomide, low dose	Adjuvant (post-RFA)	China

START NCT00990860	II	Sorafenib + TACE	BCLC B	Taiwan

NCT00855218	II	Sorafenib + TACE Placebo + TACE	BCLC B	International

COTSUN NCT00919009	II	Sorafenib + TACE	TNM III/IVa	Korea

NCT00576199	II	Bevacizumab	Pre- and Post-TACE	HK

JLOG 0901 NCT00933816	I/II	Sorafenib + fluorouracil/platinum HAI	Not suitable for resection, ablation, TACE	Japan

NCT00293436	I/II	Erlotinib + celecoxib	Adjuvant (post-resection, -TACE, or -RFA), high-risk	US

BSC, best supportive care; ECOG, Eastern Cooperative Oncology Group; HAI, hepatic arterial infusion; HK, Hong Kong; HOG, Hoosier Oncology Group; JLOG, Japan Liver Oncology Group; RFA, radiofrequency ablation; TACE, transarterial chemoembolization; US, United States; VEGF, vascular endothelial growth factor

*Anti-insulin-like growth factor receptor-1 monoclonal antibody

The combination of sorafenib and chemotherapy has been investigated in phase II trials. A randomized phase II trial found superior outcomes with the combination of sorafenib plus doxorubicin compared to placebo plus doxorubicin [57]. Median progression-free and overall survival times were 6.9 months and 13.8 months in the sorafenib arm compared to 2.8 months and 6.5 months in the placebo arm, respectively. The combination was associated with a 21% incidence of left ventricular dysfunction, though mostly of grade 1 or 2 severity. The SECOX trial evaluated sorafenib plus capecitabine and oxaliplatin [58]. Response was observed in 14% with stable disease in 61%. Median time to progression (TTP) was 7.1 months and median survival was 10.2 months. Toxicities included HFSR, diarrhea, and neutropenia. When sorafenib was paired with metronomic tegafur/uracil (UFT; 125 mg/m^2 ^twice daily), the combination led to overall response and stable disease rates of 6% and 51%, respectively [59]. Median progression-free survival was 3.7 months and median survival was 7.4 months. The most common grade 3 or 4 adverse events were fatigue (15%), HFSR (9%), and bleeding (8%).

Sunitinib has been evaluated at various doses and schedules. The SAKK 77/06 trial utilized sunitinib 37.5 mg/day continuously in 45 Swiss patients [60]. Median progression-free survival (PFS) was 2.8 months and median survival was 9.3 months. The most frequent grade 3/4 toxicities were fatigue in 24% and thrombocytopenia in 18%. Two US studies evaluated sunitinib 37.5 mg daily for 4 weeks every 6 weeks [61,62]. Response rates were 3%-6% and stable disease rates were 35%-47%. One study reported PFS and survival; median PFS was 4.0 months and median survival was 9.9 months. The most common grade 3/4 toxicities were fatigue and elevated liver function tests. A study in Europe and Asia that evaluated high-dose sunitinib (50 mg daily for 4 weeks every 6 weeks) found similar response and stable disease rates but higher toxicity with four grade 5 events [63].

Other multiple receptor tyrosine kinase inhibitors that target VEGF under investigation include brivanib, linifanib (formerly ABT-869), vandetanib, and pazopanib. Brivanib inhibits VEGF and fibroblast growth factor; a phase II trial showed median survival of 10 months in treatment-naive patients [64] and a 58% stable disease rate in patients who failed one prior antiangiogenic therapy [65]. The most frequent grade 3/4 toxicities were hyponatremia (41%), fatigue (16%), and AST elevation (19%) [64]. Linifanib inhibits VEGF and PDGF receptor tyrosine kinases. A phase II study (n = 44; 84% treatment-naïve) showed a response rate of 7%, median PFS of 3.7 months and median survival of 9.3 months [66]. Toxicities are consistent with anti-VEGF agents. A phase II, placebo-controlled study of vandetanib, which targets VEGFR, EGFR, and RET signaling, showed activity in HCC but failed to meet its primary endpoint of tumor stabilization in a Taiwanese trial [67]. A phase I dose-ranging study of pazopanib, which inhibits VEGF, PDGF, and c-kit, showed evidence of activity [68].

Phase II trials of erlotinib plus bevacizumab are promising. In 16 previously untreated patients, the combination led to a median TTP of 2.3 months and median survival of 13.7 months [69]. In 40 patients, 73% of whom were previously untreated, the response rate was 25%, median PFS was 9.0 months, and median survival was 15.7 months [70]. In 58 patients, 76% of whom were previously untreated, median PFS times were 8.8 months in patients with no prior therapy, 7.9 months in patients previously treated with sorafenib, and 6.6 months in those previously treated with therapy other than sorafenib [71]. Corresponding median survival times were 15.6 months, 13.3 months, and 14.4 months. In all studies, adverse events were consistent with the individual drug profiles.

## Asian Panel Opinions on Clinical Trial Design

In 2008, the American Association for the Study of Liver Diseases (AASLD) published a framework for clinical trial design in HCC [7]. During the current expert panel meeting, participants provided their views about clinical trial design from an Asian perspective. These views are outlined in Table 2.

**Table 2:** Eastern Asian Panel's Opinions on Clinical Trial Design Aspects

Design Aspect	Panel Opinion
*Patient Population*	

Diagnosis	• Agree with AASLD recommendations[7] - pathological confirmation OR noninvasive criteria per AASLD guidelines

Target population	• BCLC stage is acceptable, but clinical protocols must account for portal vein involvement and liver function • Treatment options for CP B/C are needed; CP B/C (ECOG PS 0 only) is an ideal population to study in advanced/metastatic HCC

Liver function	• Agree with AASLD recommendations[7]; however, trials should separately include and/or evaluate patients based on presence of cirrhosis *or *liver function grade.

Stratification	• Stratification by viral etiology is important in trials conducted within Eastern Asia • Stratification by use of antivirals should also be considered • Protocols should standardize antiviral therapy and include appropriate monitoring parameters

*Treatment*	

Control arm for RCTs	• Heterogeneity in TACE/TAE practices must be addressed • Placebo-controlled trials are feasible in unresectable disease, especially for those in whom locoregional therapy is indicated, pending maturity of post-TACE sorafenib data • AASLD recommendation for sorafenib as comparator in advanced disease [7] is not necessarily reflective of real-world use in Eastern Asia at this time due to high cost and intolerable side effects

*Phase-specific Clinical Trial Recommendations*	

Phase I	• Consider conducting Asia-specific phase I trials due to the potential for PK/PD differences between Asian and Western populations; however, Asian phase I trials may not be necessary for all targeted agents • Population • CP-A or CP score up to 7-8 (subgroup of CP-B) would be feasible for standard phase I trials • CP-B with score 8-9 and CP-C could be enrolled in phase I trials testing agents at lower doses

Phase II	• For first-line studies in advanced HCC, AASLD recommendation for sorafenib [7] is not necessarily reflective of real-world use in Eastern Asia at this time due to high cost and intolerable side effects • Agents demonstrated effective for second-line use in phase II trials (not necessarily phase III trials) can be compared to sorafenib in first-line studies

Phase III	• OS endpoint will soon no longer be appropriate in advanced disease with the introduction of multiple lines of therapies; PFS may be a surrogate but it is necessary to evaluate correlation with OS (ie, as what was done in colorectal cancer) • In unresectable disease, the most appropriate endpoint is unknown due to difficulty distinguishing recurrence from second primary in the liver and unreliability of RECIST; time to development of new lesion is a possible endpoint • Non-inferiority trials are acceptable if new agents have potential for less toxicity

AASLD, American Association for the Study of Liver Diseases; BCLC, Barcelona Clinic Liver Cancer; CP, Child-Pugh; OS, overall survival; PFS, progression-free survival; PK/PD - pharmacokinetic/pharmacodynamic; RCT, randomized controlled trial; RECIST, Response Evaluation Criteria in Solid Tumors; TACE/TAE, transarterial chemoembolization/transarterial embolization

The Asian panel also provided additional insights into clinical trial issues specific to disease stage. The panel noted a great need for trials in resectable disease. The panel felt that testing compounds in the adjuvant setting before establishing efficacy in the metastatic setting is possible, citing positive phase II adjuvant results with muparfostat (formerly PI-88) [72] and noting the need for effective therapies in this setting. The panel also expressed interest in chemoprevention with sorafenib and other agents after resection or local ablation. In unresectable disease, especially where locoregional therapy is indicated, placebo-controlled trials remain feasible, though the panel acknowledged opportunities are limited. In this setting, it may be beneficial to limit enrollment to patients who experience a maximal response after TACE based on modified EASL criteria [73]. Such a requirement would facilitate identification of subsequent disease progression across patients. However, additional research is necessary to identify the best clinical endpoints in this setting. Because it remains difficult to differentiate recurrent disease from a second primary cancer, time to development of a new lesion may be an appropriate outcome in this setting. Finally, in the advanced/metastatic setting, the panel felt that developing new agents in the second-line setting is warranted.

## Summary

Hepatocellular carcinoma is a disease of variable incidence and etiology that is managed differently worldwide. This expert panel has identified key areas that need to be addressed to facilitate clinical trials in Asia. Stratification by viral etiology is desirable within Asia and by region in global trials. Antiviral therapy should also be considered as a stratification factor and incorporated into HCC management in trials. The panel agreed with AASLD that histological diagnosis is not required for trial entry. Staging and treatment plans vary significantly. The panel felt BCLC staging is acceptable for trials as long as portal vein hypertension can be measured and defined with non-invasive standardized methodology and liver disease is further evaluated. Consensus in treatment must be sought to allow multi-national trials and it must be recognized that first-line sorafenib is not largely feasible in Asia. Finally, Asian nations must be urged to participate in clinical trials, many of which are ongoing, to advance new treatment options in this challenging disease.

## Competing interests

Junji Furuse has received honoraria from Eli Lilly, Taiho, Bayer, and Eisai, as well as research funding from Taiho. Winnie Yeo has received honoraria from Pfizer, AstraZeneca, Bristol Squibb Meyer, MSD, Roche and GlaxoSmithKline as well as research funding from Novartis. Hanlim Moon and Ee-Min Yeoh are full-time employees of GlaxoSmithKline and hold employee-restricted shares not exceeding GBP 15,000.

## Authors' contributions

All authors contributed equally to the writing of this review. All authors read and approved the final review.

## Pre-publication history

The pre-publication history for this paper can be accessed here:

http://www.biomedcentral.com/1471-2407/10/620/prepub
